# Improved 3D late gadolinium enhancement (LGE) imaging with dynamic-TI in patients with persistent atrial fibrillation

**DOI:** 10.1186/1532-429X-16-S1-P6

**Published:** 2014-01-16

**Authors:** Jennifer Keegan, Peter Gatehouse, Shouvik Haldar, Ricardo Wage, Sonya V Babu-Narayan, David Firmin

**Affiliations:** 1 Royal Brompton Hospital, London, UK; 2 Imperial College, London, UK

## Background

High resolution 3D late gadolinium enhancement (LGE) imaging prior to RF ablation of atrial fibrillation (AF) is performed with single R-wave gating to reduce lengthy acquisition times [1]. However, this increases the sequence sensitivity to RR interval variations and missed cardiac triggers which cause variable magnetization recovery between sequence repeats, leading to ghosting of blood pool and unsuppressed fat, together with poor myocardial nulling. 3D LGE image quality in AF is consequently often poor. We have developed a dynamic inversion recovery (dynamic-TI) 3D LGE sequence which minimises variations in the longitudinal magnetisation (Mz) of myocardium throughout the acquisition [2] and have performed a study to assess its efficacy in 17 patients in persistent AF.

## Methods

The dynamic-TI algorithm adjusts the inversion time automatically from beat-to-beat based on the time since the last sequence repeat [2]. Navigator-gated 3D LGE imaging (32-36 slices, 1.5 × 1.5 × 4 mm, reconstructed to 64-72 slices, 0.7 × 0.7 × .2 mm) with single R-wave gating was performed on a Siemens Avanto 1.5T scanner 15 minutes after gadolinium administration in 17 patients with persistent AF. Acquisitions were performed with and without the dynamic-TI algorithm in random order. Image quality was scored (0 = non-diagnostic, 1 = poor, 2 = fair, 3 = good) and results with and without dynamic-TI compared (paired Wilcoxon analysis). Blood pool signal-to-noise ratio (SNR) and blood-myocardium contrast-to-noise ratio (CNR) in acquisitions with and without dynamic-TI were also compared (paired t-testing). For these, the noise region of interest was positioned to include ghosting artefact from sequence repeat time variability.

## Results

**Figure 1:**
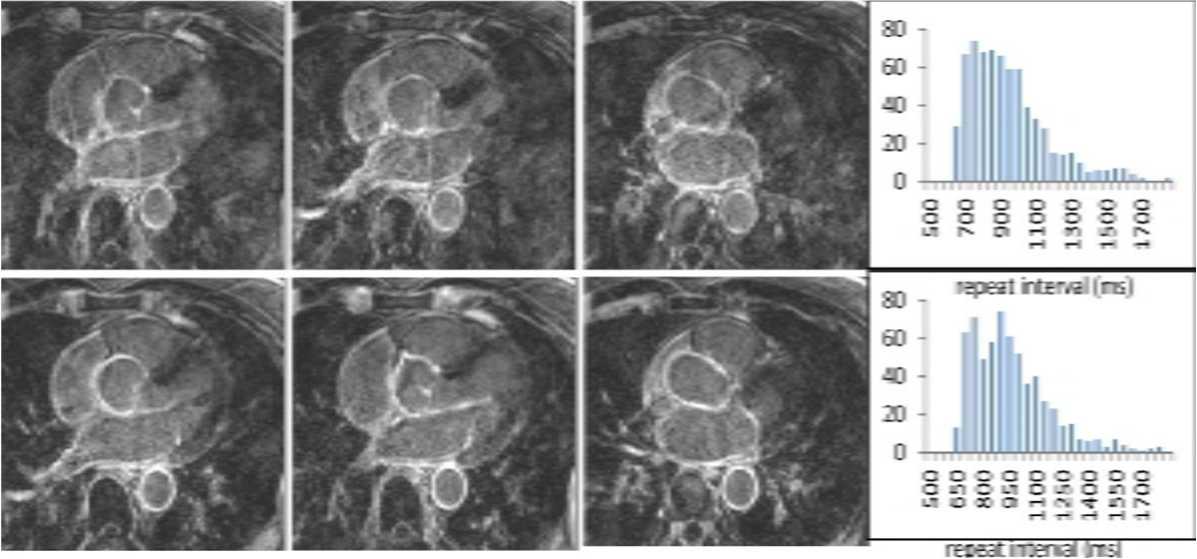
**Selected images from 3DLGE acquisitions without (top) and with (bottom) the dynamic-TI algorithm, together with histograms of the sequence repeat time intervals for each acquisition**. Sequence repeat time variability results in ghosting in the left-right phase encode direction which is reduced with the dynamic-TI algorithm.

**Figure 2:**
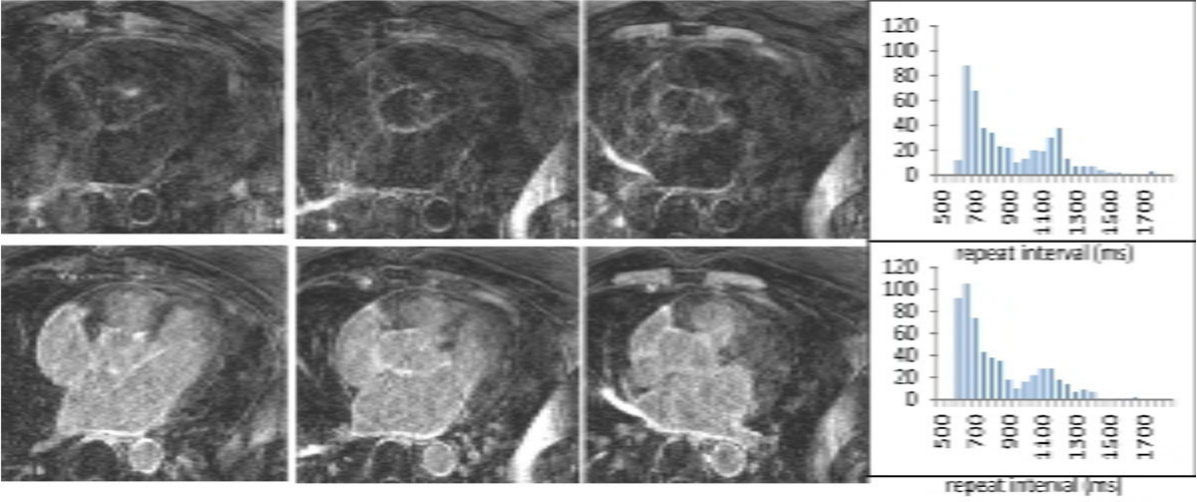
**Selected images from 3DLGE acquisitions without (top) and with (bottom) the dynamic-TI algorithm, together with histograms of the sequence repeat time intervals for each acquisition**. Missed cardiac triggers (smaller peak in histograms) result in poor nulling which is substantially improved with dynamic-TI.

Sequence repeat time variability (expressed as the standard deviation as a percentage of the mean) was the same in the acquisitions with and without dynamic-TI (26.3% +/- 6.4% vs 25.5% +/- 5.6%, p = ns). Image quality score was higher with dynamic-TI than without (2.2 +/- 0.9 versus 1.8 +/- 1.1, p < .01) as was blood-myocardium CNR (13.8 +/- 7.6 versus 8.3 +/- 6.1, p < .01) and blood pool SNR (19.6 +/- 8.5 versus 13.1 +/- 8.0, p < .01). In 8 subjects (47%), the image quality score increased with dynamic-TI, in a further 8 (47%) it stayed the same, and in 1 (6%) it decreased. In this last subject, the sequence repeat time variability was much higher in the acquisition with dynamic-TI than without (44% vs 34%). Figures 1 and 2 show patient examples demonstrating reduced ghosting and improved image quality with dynamic-TI. In Figure 2, a high percentage of missed cardiac triggers additionally resulted in poor myocardial nulling which was also improved using dynamic-TI.

## Conclusions

Dynamic adaptation of the inversion time on a beat-to-beat basis improves the image quality of 3D LGE acquisitions in patients with AF. This will assist automatic LGE quantification in the atria in this difficult patient population.

## Funding

Wellcome Trust National Institute of Health Research British Heart Foundation.
